# Maternal environmental risk factors and the development of internalizing and externalizing problems in childhood: The complex role of genetic factors

**DOI:** 10.1002/ajmg.b.32755

**Published:** 2019-08-24

**Authors:** Judith B. M. Ensink, Marleen H. M. de Moor, Mohammad Hadi Zafarmand, Sanne de Laat, André Uitterlinden, Tanja G. M. Vrijkotte, Ramón Lindauer, Christel M. Middeldorp

**Affiliations:** ^1^ Department of Child and Adolescent Psychiatry, Amsterdam Public Health Research Institute Amsterdam UMC, Location Academic Medical Center, University of Amsterdam Amsterdam The Netherlands; ^2^ Academic Center for Child and Adolescent Psychiatry De Bascule Amsterdam The Netherlands; ^3^ Department of Clinical Epidemiology, Biostatistics and Bioinformatics, Amsterdam Public Health Research Institute Amsterdam UMC, Location Academic Medical Center, University of Amsterdam Amsterdam The Netherlands; ^4^ Clinical Child and Family Studies, Amsterdam Public Health Research Institute VU University Amsterdam The Netherlands; ^5^ Department of Public Health, Amsterdam Public Health Research Institute Amsterdam UMC, Location Academic Medical Center, University of Amsterdam Amsterdam The Netherlands; ^6^ Youth Health Care GGD Hart voor Brabant 's‐Hertogenbosch The Netherlands; ^7^ Tranzo, Tilburg School of Social and Behavioral Sciences Tilburg University Tilburg The Netherlands; ^8^ Department of Epidemiology Erasmus Medical Center Rotterdam The Netherlands; ^9^ Child Health Research Centre University of Queensland Brisbane Queensland Australia; ^10^ Child and Youth Mental Health Service Children's Health Queensland Hospital and Health Service Brisbane Queensland Australia; ^11^ Biological Psychology VU University Amsterdam The Netherlands

**Keywords:** children, early life stress, gene–environment correlation, gene–environment interaction, psychopathology

## Abstract

The development of problem behavior in children is associated with exposure to environmental factors, including the maternal environment. Both are influenced by genetic factors, which may also be correlated, that is, environmental risk and problem behavior in children might be influenced by partly the same genetic factors. In addition, environmental and genetic factors could interact with each other increasing the risk of problem behavior in children. To date, limited research investigated these mechanisms in a genome‐wide approach. Therefore, the goal of this study was to investigate the association between genetic risk for psychiatric and related traits, as indicated by polygenetic risk scores (PRSs), exposure to previously identified maternal risk factors, and problem behavior in a sample of 1,154 children from the Amsterdam Born Children and their Development study at ages 5–6 and 11–12 years old. The PRSs were derived from genome‐wide association studies (GWASs) on schizophrenia, major depressive disorder, neuroticism, and wellbeing. Regression analysis showed that the PRSs were associated with exposure to multiple environmental risk factors, suggesting passive gene–environment correlation. In addition, the PRS based on the schizophrenia GWAS was associated with externalizing behavior problems in children at age 5–6. We did not find any association with problem behavior for the other PRSs. Our results indicate that genetic predispositions for psychiatric disorders and wellbeing are associated with early environmental risk factors for children's problem behavior.

**Abbreviations**
DASS: depression anxiety stress scales
GWAS: genome‐wide association study
GxE: gene–environment interaction
PRSs: polygenic risk score(s)
rGE: gene–environment correlation
SDQ: strengths and difficulties questionnaire
STAI: the state–trait anxiety inventory

## 1 INTRODUCTION

Longitudinal studies that followed children from pregnancy onward have consistently shown that exposure to maternal prenatal adverse environmental factors is associated with the development of cognitive, externalizing, and internalizing problems in children. For instance, exposure to maternal smoking during pregnancy, use of alcohol during pregnancy, maternal age at gestation, and high rates of anxiety and distress in the mother are related to adverse outcomes later in childhood (Buss, Davis, Hobel, & Sandman, 2011; Loomans et al., 2011; MacKinnon, Kingsbury, Mahedy, Evans, & Colman, 2018; Madigan et al., 2018; O'connor, Heron, Golding, & Glover, 2003; Van den Bergh, Van Calster, Smits, Van Huffel, & Lagae, 2008). Besides exposure to these adverse environmental risk factors, genetic risk is associated with the development of problem behavior in childhood. The influence of genetic risk on internalizing and externalizing problems in children is studied intensively with twin and family studies. Heritability estimates vary from 20 to 50% for internalizing problems to over 60% for externalizing problems (Hannigan, Walaker, Waszczuk, McAdams, & Eley, 2017).

It is well possible that the genetic factors associated with the development of problem behavior, are also related to the early environment risk factors linked to the development of problem behavior, that is, gene–environment correlation (rGE). For example, when a mother has a genetic vulnerability to experience distress, this can result in the exposure of the child to adverse environmental influences such as maternal anxiety and depression during pregnancy as well as to the transmission of the maternal genetic vulnerability. Gene–environment interaction (GxE) may also be a part of the gene–environment interplay influencing the development of problem behavior. GxE means that a child's behavioral reaction on exposure to adverse environmental factors depends on his or her genotype. GxE and rGE are independent mechanisms but may impact the child's development simultaneously. Moreover, a GxE effect can be observed erroneously if rGE is present but not taken into account (Rutter, Moffitt, & Caspi, 2006).

To date, longitudinal studies that obtained repeated measures of problem behavior have provided limited information about how genetic factors may interact or correlate with early environmental risk. Recently a review has been published providing an overview of studies that investigated GxE in relation to prenatal stress and risk for psychiatric illness (Abbott, Gumusoglu, Bittle, Beversdorf, & Stevens, 2018). This overview concluded that exposure to prenatal environmental risk factors modifies the genetic risk for psychopathology. Some of the reported studies state that vulnerability for psychopathology increases after exposure to prenatal risk factors depending on heritable influences as in a “diathesis stress model”. Other studies report that heritable factors impact the susceptibility for prenatal environment risk for better and worse, referred to as the “differential susceptibility model”. Most of these studies described used a candidate‐gene approach examining the influence of single genetic risk variants in interaction with environmental exposures (Abbott et al., 2018). However, it is expected that genetic variation within hundreds to thousands of genes contribute to the heritability of psychopathology (Gratten, Wray, Keller, & Visscher, 2014). In addition, rGE mechanisms are often not investigated in GxE studies, but have been suggested to be of importance as well (Abbott et al., 2018). This requires alternative designs to test rGE and GxE mechanisms in relation to prenatal stress, such as the use of polygenetic risk scores (PRSs) (Gratten et al., 2014), which will likely improve the accuracy to predict the risk for the development of complex traits on an individual level compared with candidate‐gene models (Bogdan, Baranger, & Agrawal, 2018; Mistry, Harrison, Smith, Escott‐Price, & Zammit, 2017, 2018). See for more details about the construction and value of the PRS method: Middeldorp and Wray (2018).

Recent studies have shown that PRS that were based on findings from large GWA data sets based on psychiatric phenotypes such as, schizophrenia and major depressive disorder (MDD) are associated with the development of psychopathology, in children (Jansen et al., 2018; Krapohl et al., 2016; Nivard et al., 2015; Riglin et al., 2017; Trotta et al., 2016). To date PRS have rarely been applied to investigate rGE and GxE as mechanisms to explain the risk for psychopathology in childhood.

To our knowledge only one study investigated the relation between PRSs, (prenatal) environmental risk, and developmental outcomes in childhood (Krapohl et al., 2017). In this study, PRSs were based on GWAS of educational attainment, BMI, and schizophrenia. These PRSs were related to three developmental outcomes in childhood; educational achievement, inattention, and hyperactivity symptoms, and conduct problems as well as to multiple environmental risk factors related to parental characteristics, such as breastfeeding duration, parental age at birth, household income, and parental smacking. The study showed that environmental risk, already present at birth or early in life, correlates with offspring genetic vulnerabilities as expressed by all PRSs. In addition, the education‐associated PRS captured partly the covariation between parental slapping/smacking and conduct problems and hyperactivity/inattention problems. An investigation of possible GxE mechanisms between these environmental factors and PRSs was not reported (Krapohl et al., 2017).

Studies on adult outcomes have also investigated rGE as an explanation of the association with childhood environmental risk factors, such as exposure to childhood trauma (Mullins et al., 2016; Musliner et al., 2015; Peyrot et al., 2014, 2018; Trotta et al., 2016) and parenting and peer factors (Agerbo et al., 2015; Salvatore et al., 2014). These studies reported that the PRS and environmental risk factors are both related to the outcome of interest.

The most recent largest study reported rGE between the MDD based PRS and the number of stressful life events within cases with high rates of depression symptom and population‐based cohorts, however effect sizes are small (Peyrot et al., 2018). No evidence for interaction between a MDD based PRS and childhood trauma was reported (Peyrot et al., 2018). rGE was not observed for the schizophrenia‐based PRSs and childhood adversity. In the study of Trotta et al. (2016), a higher schizophrenia PRS and exposure to childhood adversities each predicted psychosis status. Nevertheless, no evidence was found for a correlation or interaction as a departure from additivity, indicating that the effect of a PRS on psychosis was not increased in the presence of a history of childhood adversity. Further research is required, but these studies suggests that the genetic heterogeneity of MDD, or schizophrenia is not attributable to genome‐wide moderation of genetic effects by childhood adversity. Previously a smaller study reported GxE for the MDD PRS, although in the opposite direction as expected. This might be best interpreted as a chance finding (Mullins et al., 2016).

Furthermore, the schizophrenia‐based PRS was related to a current schizophrenia diagnosis, socioeconomic status, and a family history of schizophrenia/psychoses (rGE). In addition the effect associated with family history of schizophrenia/psychoses was mediated through the PRS, indicating GxE. A PRS derived from a GWAS on externalizing problems predicted externalizing behavior and impulsivity traits in adolescents. Adolescent parental monitoring and peer substance use moderated the PRS to predict externalizing disorders, indicating GxE (Salvatore et al., 2014). The reported inconsistencies in the rGE and GxE studies might be explained by differences in the method of assessment (self‐report vs. interviews) and differences in the GWA discovery samples that were used to calculate the PRS. Furthermore, the sizes of target sample varied highly.

Following these findings, our aim is to further examine the association between PRS based on findings from adult GWA meta‐analyses for schizophrenia, depression, neuroticism, and wellbeing (Okbay et al., 2016; Ripke et al., 2014) with exposure to early environmental risk factors and children's problem behavior, testing both rGE and GxE mechanisms. These adult psychiatric phenotypes were used because previous studies have indicated the relevance to the child's problem behavior.

More specifically we investigated: (a) the associations of PRSs and the development of internalizing and externalizing problems in children of the Amsterdam Born Children and their Development (ABCD) cohort study at two different time points (children's age 5–6 and children's age 11–12), (b) the associations between the PRSs and maternal prenatal and childhood risk factors associated with the development of children's problem behavior, and (c) for the PRS that showed a significant association with children's problem behavior, the interaction between the PRS and the maternal prenatal and childhood risk factors on the development of problem behavior in childhood.

## 2 METHODS

### 2.1 Participants and procedure

This study is part of the ABCD study (http://www.abcd-study.nl). The ABCD study is a population‐based prospective birth cohort study investigating how factors in early life (during pregnancy and infancy) are associated with health later in life. Details of the study design are described elsewhere (Van Eijsden, Vrijkotte, Gemke, & van der Wal, 2011). In brief, between January 2003 and March 2004, all pregnant women living in Amsterdam, the Netherlands, were asked to participate in the study during their first visit to the general practitioner, midwife, or gynecologist. In total 12,373 women where approached and 8,266 returned the first questionnaire during pregnancy. Data for this study come from ABCD‐Genetic Enrichment (ABCD‐GE) study, a substudy of ethnically Dutch children. Mothers and their children were included if the child's genetic data were available (*N* = 1,154). Children's problem behavior was assessed prospectively at the age of 5–6 (Phase 3 of the ABCD study) and age 11–12 (Phase 4 of the ABCD study). Data collection consisted of mother (*N* = 1,148) and teacher (*N* = 999) reports at age 5–6, and mother reports (*N* = 816), teacher (*N* = 816), and child (*N* = 816) reports at age 11–12. The following maternal prenatal environmental risk factors were selected based on an earlier study that was conducted within the ABCD cohort (Loomans et al., 2011): maternal education, maternal smoking/use of alcohol during pregnancy, maternal age at gestation, maternal anxiety, and psychopathology. Furthermore, we included the perceived amount of distress in the mother at the moment of the measurement (child's age 5–6 and 11–12) as an environmental risk factor during childhood. The study was approved by the Institutional Review Board of the Academic Medical Center, Amsterdam, the Netherlands. All participants provided written informed consent for data collection of the behavioral and environmental assessments. Regarding the DNA collection and analysis, an opt‐out procedure was used (METC approval 2002_039#B2013531).

### 2.2 Measurements

#### 2.2.1 Maternal environmental risk factors

The maternal prenatal risk factors were assessed during the 16th week of gestation. At this time point, self‐report information about maternal education (low, middle, high), maternal age at gestation (years), maternal smoking and use of alcohol during pregnancy (ratings of amounts per day during the first weeks of gestation), and psychopathology (yes/no regarding a history of psychopathology) were obtained (Loomans et al., 2011). Maternal prenatal anxiety was assessed using the Dutch version of the state–trait anxiety inventory (STAI) (Spielberger, 1970). The 20 items about state anxiety (transient or temporarily experienced anxiety over the preceding week) were included in our questionnaire, with each item scored on a four‐point scale (0 = rarely or none of the time, 1 = some or a little of the time, 2 = occasionally or a moderate amount of the time, and 3 = most or all of the time). In addition, current maternal distress at the child's age 5–6 and current maternal distress at the child's age 11–12 were measured with the short version of the Depression Anxiety Stress Scales (DASS) (Henry & Crawford, 2005) and included as childhood environmental risk factors. The DASS consists of 21 items designed to assess depression, anxiety, and stress in adults. Answers range from 0 (not at all) to 3 (most of the time) with higher scores indicating increasing anxiety, depression, or stress.

#### 2.2.2 Children's internalizing and externalizing problems

Children's mental health was reported by their mothers and primary school teachers using the strengths and difficulties questionnaire (SDQ) at age 5–6 and age 11–12. In addition, at age 11–12, children filled in the self‐report questionnaire of the SDQ. The SDQ is a short screening questionnaire suitable for 2‐ to 17‐year olds. The questionnaire consists of 25 items, with positive and negative statements, which cluster in five scales: emotional symptoms, conduct problems, hyperactivity/inattention problems, peer relationship problems, and prosocial behavior. The internalizing problem scale is based on emotional symptoms plus peer relationship items and the externalizing problem scale is based on conduct plus hyperactivity/inattention items (Goodman, Lamping, & Ploubidis, 2010).

### 2.3 Genotyping and PRS

During the 5‐year health check‐up of the children (2008–2010) blood was collected with a finger prick. DNA was extracted from the dried blood spots and samples were genotyped, using the Illumina Human Core Exom Beadchip (Illumina, San Diego, California). The Illumina Human Core Exom Beadchip included over 540,000 genetic markers. Genotyping was performed in April 2014 by the Human Genomics Facility at Erasmus MC, Rotterdam (http://www.glimdna.org). Participants were excluded based on: genetic quality control (*n* = 25, call rate <95%; heterozygosity (±3 *SD* of the mean), phenotype–genotype gender mismatch (*n* = 20), and relatedness (*n* = 1, proportion of IBD in PLINK >0.2). This resulted in 1,154 children with quality controlled GWAS data. Before imputation, SNPs were excluded if they had high levels of missing data (SNP call rate <95%), strong departures from Hardy–Weinberg equilibrium (*p* < 1 × 10^−6^), or low minor allele frequencies (<1%), leaving 277,644 SNPs for imputation. Genetic markers were imputed (total SNPs after imputation 27,448,454) using the IMPUTE2 software and the 1000 Genomes References Panel (phase 1 release v3, build 37).

Polygenic scores were based on the summary statistics available for schizophrenia (Ripke et al., 2014), depression, neuroticism, and wellbeing GWA meta‐analyses (Okbay et al., 2016). They were calculated using LDpred. LDpred is a Bayesian approach that calculates a PRS, after adjusting for linkage disequilibrium (LD), enabling the use of all SNP information across the genome to calculate the PRS. Shortly, LD adjustment is performed by calculating the LD information for a given radius of the genome in the data set, and by using that LD information to weigh the summary statistics (Vilhjálmsson et al., 2015). These weighted effect sizes were then used in PLINK2 to construct PRS (Purcell et al., 2007). For each summary statistic, we included SNPs with a threshold of *r* ^2^ > .9 and a minor allele frequency above 5%. The PRSs were transformed to unit variance and mean centered within our cohort. First, we created PRS using different priors (0.6, 0.7, 0.8, 0.9, and 1). In the multiple hierarchic regression model, we used only the PRSs based on the prior 1, as this was the prior that yielded the largest *r* ^2^ in general.

### 2.4 Statistical analysis

IBM SPSS (version 24.0) was used for all statistical analyses. To control for outliers, reduce skewness and improve normality, linearity, and homoscedasticity of residuals a square root transformation was used on all continuous problem behavior and environmental risk variables. First, we tested whether the PRS predicted the development of children's problem behavior with linear regression analysis. Second, we tested the association between the PRS and the maternal prenatal and childhood environmental risk factors with linear or logistic regression. We conducted a univariable linear regression analysis for the continuous risk factors, that is, maternal age at gestation, maternal anxiety, and the current maternal distress (at child's age 5–6 or 11–12). We conducted a univariable logistic regression analysis for maternal smoking (yes vs. no) and use of alcohol (yes vs. no), maternal education (low/middle vs. high) and for self‐report of psychopathology (yes vs. no). Third, we tested whether the PRS explained additional variance regarding the child's outcomes above the prediction by our environmental variables with a hierarchical regression analysis (enter method). We included age, and gender in Model 1, the environmental risk factors in Model 2, and the PRS was added in Model 3. If the main effects of the PRSs were still significant after controlling for the environmental predictors in Model 3, we subsequently tested whether there was interaction between the PRS and the environmental risk factors. All outcomes were tested separately for children's age 5–6 and children's age 11–12, and for the different raters. To correct for multiple testing in the correlated outcome variables, we estimated the effective number of phenotypes studied using Matrix Spectral Decomposition “MatSpD” (https://gump.qimr.edu.au/general/daleN/matSpD/). MatSpD calculates a threshold for statistical significance based on the independent number of outcome variables taking into account the correlation matrix of all variables across the different time points, yielding a *p* value <.005 to be considered statistically significant (Nyholt, 2004).

## 3 RESULTS

### 3.1 Sample characteristics

Demographic and clinical characteristics of the participating mothers and children are shown in Table S1. The children had a mean age of 5.11 (SD 0.2) at time point 1 (age 5–6) and of 11.55 (SD 0.3) at time point 2 (age 11–12). At both time point's gender was almost equally distributed and all children had an ethnic Dutch background (which was a selection criterion for genotyping). Bivariate correlations between mother, teacher, and child ratings at both measurements are presented in Table S2, and ranged between 0.10 and 0.58 across informant and time for internalizing problem behavior and between 0.28 and 0.62 for externalizing behavior. The PRS for schizophrenia, depression, neuroticism, and wellbeing all correlated significantly with each other and in the expected directions (see Table S3).

### 3.2 PRS and internalizing and externalizing problems in childhood

Table 1 presents the relationships between the PRS for schizophrenia, depression, neuroticism, and wellbeing at one hand with internalizing and externalizing problems in childhood on the other hand. Only the association between the PRS for schizophrenia and children's externalizing behavior problems reported by the mother at children's age 5–6 was significant after multiple testing correction (*β* = 0.097, *R* ^2^ = .011, *p* = .001, see Table 1).

**Table 1:** Standardized regression coefficients for the univariate linear regression analyses with childhood problem behavior predicted by the PRS for schizophrenia, depression, neuroticism, and wellbeing

			PRS schizophrenia				PRS depression				PRS neuroticism				PRS wellbeing			
			*β*	*SE*	*p*‐Value	*R* ^2^	*β*	*SE*	*p*‐Value	*R* ^2^	*β*	*SE*	*p*‐Value	*R* ^2^	*β*	*SE*	*p*‐Value	*R* ^2^
Problem behavior age 5–6	Mother	Int	0.066	0.015	.026	.004	0.077	0.014	.009	.006	0.049	0.015	.098	.003	−0.037	0.015	.205	.002
		Ext	**0.097**	**0.020**	.001*	.011	0.049	0.020	.093	.004	0.018	0.020	.542	.002	−0.043	0.020	.149	.003
	Teacher	Int	0.009	0.020	.787	.001	−0.026	0.019	.409	.001	0.013	0.019	.671	.001	−0.013	0.019	.693	.001
		Ext	0.048	0.015	.133	.002	0.033	0.025	.300	.001	0.040	0.025	.210	.002	−0.046	0.025	.147	.002
Problem behavior age 11–12	Mother	Int	−0.031	0.026	.382	.001	0.063	0.023	.073	.004	0.046	0.024	.189	.002	0.000	0.024	1.00	–
		Ext	0.012	0.028	.739	–	0.045	0.026	.201	.002	0.071	0.026	.044	.005	−0.032	0.026	.367	.001
	Teacher	Int	−0.043	0.010	.286	.002	0.035	0.009	.385	.002	0.011	0.010	.265	–	−0.017	0.009	.678	.001
		Ext	0.069	0.013	.086	.005	0.061	0.012	.127	.004	0.075	0.013	.060	.006	−0.067	0.012	.096	.005
	Child	Int	0.040	0.022	.235	.003	0.072	0.021	.033	.006	0.092	0.021	.007	.009	−0.074	0.021	.028	.006
		Ext	0.024	0.037	.983	–	0.034	0.023	.315	.001	0.027	0.013	.426	.001	−0.046	0.023	.168	.002

*Note*: Polygenetic risk scores that are reported here were calculated based on a Gaussian Prior of 1. Gender was included as a covariate. Bold values indicate all significant findings.

Abbreviations: Ext, SDQ score for externalizing problem behavior; Int, SDQ score for internalizing problem behavior; PRS, polygenetic risk score; SDQ, strengths and difficulties questionnaire.

*Significant after multiple correction (based on *p <* .005).

### 3.3 PRS and maternal environmental risk factors

Table 2 presents the relationships between the PRS for schizophrenia, depression, neuroticism, and wellbeing with the environmental risk factors. The PRS for schizophrenia was negatively associated with maternal education, use of alcohol during pregnancy and age of the mother at gestation, indicating that higher polygenetic risk for schizophrenia is associated with lower education, decrease of alcohol use during pregnancy, and younger maternal gestational age (Table 2). In addition, the PRS for depression was positively associated with maternal prenatal anxiety (high PRS score is associated with higher maternal prenatal anxiety score), and current rates of distress in the mother at children's age 5–6 (high PRS score is associated with a higher distress score). The PRS for neuroticism is positively related to maternal prenatal anxiety (high PRS score is associated with higher maternal prenatal anxiety scorer) and negatively associated with the risk of alcohol use during pregnancy (higher PRS score is associated with less alcohol consumption).

**Table 2:** Standardized regression coefficients for the linear and logistic regression analyses with maternal environmental risk factors predicted by the PRS for schizophrenia, depression, neuroticism, and wellbeing

	PRS schizophrenia				PRS depression				PRS neuroticism				PRS wellbeing			
	*β*	*SE*	*p*‐Value	*R* ^*2*^	*β*	*SE*	*p*‐Value	*R* ^*2*^	*β*	*SE*	*p*‐Value	*R* ^*2*^	*β*	*SE*	*p*‐Value	*R* ^*2*^
Age at gestation	−0.160	0.121	.000*	0.26	−0.001	0.123	.968	–	−0.029	0.123	.324	.001	0.072	0.122	.015	.005
Maternal anxiety during pregnancy	0.069	0.023	.018	.004	0.110	0.023	.000*	.012	0.093	0.023	.002*	.009	−0.066	0.023	.025	.004
Maternal distress at child's age 5–6	0.009	0.040	.816	–	0.108	0.037	.002*	.012	0.093	0.037	.008	.009	−0.041	0.037	.249	.002
Maternal distress at child's age 11–12	0.009	0.020	.804	–	0.091	0.019	.010	.008	0.079	0.019	.024	.006	−0.039	0.019	.262	.002
	OR	*SE*	*p*‐Value	*R* ^*2*^	OR	*SE*	*p*‐Value	*R* ^*2*^	OR	*SE*	*p*‐Value	*R* ^*2*^	OR	*SE*	*P*‐Value	*R* ^*2*^
Smoking during pregnancy	1.29	0.095	.007	.013	1.18	0.102	.103	.005	1.16	0.100	.135	.004	0.766	0.099	.007	.013
Alcohol during pregnancy	0.811	0.066	.001*	.013	0.859	0.064	.017	.007	0.833	0.064	.005*	.010	1.11	0.064	.104	.003
Prenatal psychopathology	1.11	0.071	.148	.003	1.17	0.073	.037	.006	1.15	0.073	.056	.005	0.885	0.073	.093	.004
Education	0.759	0.068	.000*	.021	0.857	0.068	.024	.007	0.880	0.068	.061	.005	1.20	0.068	.010	.009

*Note*: Polygenetic risk scores that are reported here were calculated based on a Gaussian Prior of 1.We conducted a univariable linear regression analysis for the continuous risk factors, that is, maternal age at gestation, maternal anxiety, and the current maternal distress (at child's age 5–6 or 11–12). We conducted a univariable logistic regression analysis for maternal smoking (yes vs. no), maternal use of alcohol (yes vs. no), maternal education (low/middle vs. high), and for self‐report of psychopathology (yes vs. no). For the linear regression *β* coefficients are reported, for the logistic regression the odds ratio (OR), and Nagelkerke *R* ^2^ is reported.

Abbreviation: PRS, polygenetic risk score.

*Significant after multiple correction (based on a *p* value of .005).

### 3.4 Hierarchical regression analysis PRS and behavioral outcomes

To estimate the additional predictive value of each polygenic score in relation to the development of problem behavior, we performed a hierarchical multiple regression analysis. The proportions of variance in internalizing and externalizing problems explained by environmental risk factors ranged between 2.5 and 11.7%, whereas the proportions of variance additionally explained by genetic risk was at most 0.06% (see Table S4). Results showed that after correction for multiple testing, the PRS did not have additive predictive value in the prediction of behavioral outcomes in addition to the environmental risk factors. Because of the limited predictive value of PRS on problem behavior after including the environmental risk factors, we did not further investigate an interaction effect between the PRSs and exposures to maternal prenatal environmental risk factors on childhood internalizing and externalizing problems.

## 4 DISCUSSION

Our study investigated the associations between polygenetic and environmental risk factors and the development of internalizing and externalizing problems in children aged 5–6 and 11–12 years old. Our results confirm that prenatal and childhood maternal environmental risk are associated with the development of problem behavior in childhood. We find limited evidence for the association between genetic factors, measured with PRSs based on adult psychiatric and related traits, and the development of problem behavior in childhood. Rather, the PRSs are associated with the maternal environmental risk factors. In other words, the genetic make‐up of the child, as expressed by the PRS, is associated with the environment the child is exposed too, in this case part of the prenatal and childhood environment provided by the mother. These results indicate rGE as a possible mechanism explaining part of association between the risk factors and problem behavior in childhood. This likely mainly represents passive rGE rather than reactive or active rGE, given that the PRS are also already correlated with the prenatal variables. However, current maternal distress was also found associated with PRS, which could be due to reactive rGE, that is, the distress in the mother being a reaction to the child's problem behavior. After controlling for the risk factors, polygenetic risk did not explain additional variance in childhood problem behavior, and we therefore did not test for GxE anymore.

Our results are in line with an earlier study on rGE (Krapohl et al., 2017) that reported significant relationships between children's PRSs based on schizophrenia, BMI, and education attainment with family environmental risk factors, such as paternal age, maternal smoking during pregnancy, and household income (Krapohl et al., 2017). In contrast with other studies, our study found hardly any association between the PRS and childhood problem behavior (Baselmans et al., 2019; Nivard et al., 2015; Peyrot et al., 2018; Riglin et al., 2017). An exception is the significant association for the PRS of schizophrenia with externalizing problems at age 5–6 reported by the mother, which has also been found by Jansen et al. (2018) in an independent but comparable birth cohort from the Netherlands. Similar to the results of this study, the effect of the schizophrenia PRS was no longer significant when the children were older, nor did it remain after controlling for environmental risk. The lack of replication of stronger findings for the positive association between PRSs and childhood emotional and behavioral problems may possibly be explained by our relatively small sample. However, the study of Dudbridge (2013) suggests that a PRS explaining between 0.01 and 0.6% of variance, with 80% power could arise in smaller sample sizes (>800).

Given the study design our results cannot disentangle whether the maternal genetic factors influence the environment which in turn influences the child's behavior (environmental mediation of genetic effects) or whether the genetic factors independently influence both the environment and the child's behavior (i.e., genetic pleiotropy). We are also limited by use of self‐report questionnaires to measure predictors and outcomes. In line with other studies that also used the SDQ, children's self‐report, parent and teacher ratings are only modestly correlated (Becker, Hagenberg, Roessner, Woerner, & Rothenberger, 2004). At the same time, it can also be seen as a strength of the study that child problem behavior was based on multiple informants and conducted at multiple time points in different settings. Other strengths of the study are that we used the results of relatively powerful GWA studies, although these results have in the meanwhile been superseded by other GWA studies (Baselmans et al., 2019; Pardiñas et al., 2018; Wray et al., 2018). We also applied the LDpred method (Vilhjálmsson et al., 2015) for calculating PRSs. Because this method includes all genetic markers across the genome without preselecting markers using a *p*‐value threshold, it is thought that the PRS that are calculated with this method are more accurate predictors of complex traits in comparison with traditional PRS methods developed by International Schizophrenia Consortium et al. (2009). Lastly, the LDpred algorithm used in this study has improved prediction accuracy compared to traditional methods. However, a recent study has suggested that the method may still provide an underestimation of the variance explained. Another method to calculate a PRS with reliable corrections for LD, that is, nonparametric shrinkage may further improve the predication accuracy (Chun et al., 2019). A final strength is that our sample consisted of a homogeneous group of ethnically Dutch children, hence population stratification is not likely to have affected our outcomes.

For future studies, we recommend to include information from multiple raters, and use additional measurements, such as item response methods. With this information we might be able to construct more reliable behavior problem phenotypes. Also, the accuracy of the PRS itself will be improved by further increasing the sample size of the GWA meta‐analyses that serve as the discovery cohorts for polygenic risk prediction efforts. Other advanced approaches for calculating PRS could further improve the accuracy of the predictions. For example, by the incorporation of additional data based on biological mechanisms that are proposed to affect the development of problem behavior, such as gene transcription information (Bogdan et al., 2018; Pratt & Hall, 2018). Furthermore, given that more and more child cohorts are enriched with genome‐wide genetic data nowadays, it becomes feasible to study polygene‐environment interplay mechanisms in explaining childhood problem behavior by meta‐analytic techniques. Lastly, cohorts with data available from multiple members of a family (e.g., parents and their offspring) can be useful for more in depth analyses of transgenerational effects. Such a design could provide more insight in the effects of transmitted alleles of the parents to their offspring and their relation to environmental risk, but also enables us also to better understand the relation between nontransmitted alleles and their impact on environmental risk factors, such as the nurturing environment provided by the parents and other relatives that are likely to affect the child's development (Kong et al., 2018).

In conclusion, this study indicates that genetic predispositions for psychiatric disorders and wellbeing are associated with early environmental risk factors for children's problem behavior, pointing to rGE mechanisms. A child's genetic predisposition for the development of psychopathology is related to a child's risk to be exposed to environmental risk factors, already prenatally, together they might further explain the development of problem behavior during childhood. These results may in the future be valuable to select children to test prevention or intervention strategies.

## CONFLICT OF INTEREST

The authors declare no potential conflict of interest.

## Supporting information


**TABLE S1** Demographic and clinical characteristics of participating children and their mothers
**Table S2**: Correlations between mother report. Teacher report and child's report scores on the SDQ
**Table S3**: Pearson's correlations between the four polygenetic risk scores
**Table S4**: Results of the hierarchical regression analyses with the environmental risk factors and the PRSs for Schizophrenia. Depression. Neuroticism and Wellbeing predicting child internalizing and externalizing symptoms at ages 5/6 and 11/12.
