# Supplementary material for: Maternal environmental risk factors and the development of internalizing and externalizing problems in childhood: The complex role of genetic factors
Source: Am J Med Genet B Neuropsychiatr Genet. 2019 Aug 24;183(1):17–25. doi: 10.1002/ajmg.b.32755 (PMC6916208; doi:10.1002/ajmg.b.32755)
Supplement: Supplementary file 1 — TABLE S1 Demographic and clinical characteristics of participating children and their mothers Table S2: Correlations between mother report. Teacher report and child's report scores on the SDQ Table S3: Pearson's correlations between the four polygenetic risk scores Table S4: Results of the hierarchical regression analyses with the environmental risk factors and the PRSs for Schizophrenia. Depression. Neuroticism and Wellbeing predicting child internalizing and externalizing symptoms at ages 5/6 and 11/12. [file AJMG-183-17-s001.docx]

Supporting information

sTABLE 1: Demographic and clinical characteristics of participating children and their mothers

|  | Child’s age 5/6(n=1154) | | Child’s age 11/12 (n=932) | |
| --- | --- | --- | --- | --- |
| **Child’s report characteristics** | | | | |
|  | Boys (n=560) | Girls (n=594) | Boys (n=450) | Girls (n=482) |
| Age | 5.11 (0.19) | 5.14 (0.21) | 11.57 (0.32) | 11.54 (.30) |
| Internalizing problem behavior (SDQ)  Parent  Teacher report  Child’s report | 1.53 (1.85) 2.17(2.48) - | 1.38(1.65) 1.83(2.14) - | 2.77(2.97) 2.67(1.09) 2.69 (2.58) | 2.66 (2.67) 2.47 (0.91) 3.01 (2.56) |
| Externalizing problem behavior (SDQ)  Parent  Teacher report  Child’s report | 3.90 (3.09)  3.69(3.08) xxx | 2.79 (2.57) 2.80 (2.57) xxx | 4.21 (3.44) 4.55 (1.80) 5.16 (3.23) | 3.68 (1.13) 3.71 (1.15) 4.47 (2.91) |
| **Maternal characteristics** | | | | |
| Maternal age at gestation | 32.80(4.23) | 32.89 (4.09) | 33.22(3.95) | 32.90(4.02) |
| Maternal education  Low  Middle  High | 36 (6.4%) 90 (16.1%) 427(76.3%) | 45 (7.6%) 115 (19.4%) 430 (72.4%) | 20 (4.5%) 73 (16.2%) 351(78%) | 27 (5.6%)  90 (18.7%) 363 (75.3%) |
| Smoking  >1 cigarette a day  <1 cigarette a day  No | 42 (7.5%)  19 (3.4%)  499 (89.1%) | 37 (6.2%)  12 (2.0%)  545 (91.8%) | 27 (6.0%) 13 (2.9%) 410 (91.1%) | 26 (5.4%) 8 (1.7%) 448 (92.9 %) |
| Alcohol consumption  >1 glass a day  <1 glass a day  No | 8 (1.4%) 164 (29.3%) 388 (69.3%) | 3 (0.5%)  187 (31.5%)  404 (68.0%) | 6 (1.3%) 140 (31.1%) 304 (67.6%) | 1 (0.2%) 156 (32.4%) 325 (67.4%) |
| Anxiety symptoms (STAI) | 36.03 (10.14) | 35.55 (9.00) | 35.60 (9.203) | 35.84 (10.02) |
| Self-report Parental Psychopathology  Yes  No  Unknown | 141 (26.96%)  476 (85%)  3 (0.54%) | 101 (17%)  432 (72.7%)  1 (0.17%) | 112 (12.89%)  382 (84.89%) 3 (0.67%) | 83(17.22%) 351 (72.82%) 1 (0.21 %) |
| Maternal current distress (DASS21) | 7.81 (10.45) | 8.20 (10.78) | 6.87 (6.35) | 7.02 (5.40) |

Continuous variables are presented as mean (standard deviation); categorical variables are presented as frequency (percentage). SDQ Strengths and Difficulties Questionnaire; STAI State Trait Anxiety Inventory. DASS-21 Depression Anxiety and Stress Scales. For self-report of psychopathology 4 mothers reported ‘unknown’. they were not included in the further analysis.

sTABLE 2: Correlations between mother report. teacher report and child’s report scores on the SDQ

|  | | | SDQ scores at age 5/6 | | | | SDQ scores at age 11/12 | | | | | |
| --- | --- | --- | --- | --- | --- | --- | --- | --- | --- | --- | --- | --- |
|  | | | Mother report | | Teacher report | | Child’s report | | Mother report | | Teacher report | |
|  | | | INT | EXT | INT | EXT | INT | EXT | INT | EXT | INT | EXT |
| Age 5/6 | Mother report | INT | 1 | .271^**^ | .372^**^ | .137^**^ | .156^**^ | .088^**^ | .392^**^ | .192^**^ | .212^**^ | .024 |
|  |  | EXT |  | 1 | .112^**^ | .508^**^ | .142^**^ | .356^**^ | .262^**^ | .508^**^ | .192^**^ | .325^**^ |
|  | Teacher report | INT |  |  | 1 | .221^**^ | .102^**^ | .057 | .251^**^ | .121^**^ | .225^**^ | .030 |
|  |  | EXT |  |  |  | 1 | .128^**^ | .281^**^ | .181^**^ | .373^**^ | .192^**^ | .362^**^ |
| Age 11/12 | Child’s report | INT |  |  |  |  | 1 | .369^**^ | .575^**^ | .238^**^ | .300^**^ | .106^**^ |
|  |  | EXT |  |  |  |  |  | 1 | .305^**^ | .620^**^ | .220^**^ | .404^**^ |
|  | Mother report | INT |  |  |  |  |  |  | 1 | .388^**^ | .377^**^ | .159^**^ |
|  |  | EXT |  |  |  |  |  |  |  | 1 | .238^**^ | .506^**^ |
|  | Teacher report | INT |  |  |  |  |  |  |  |  | 1 | .229^**^ |
|  |  | EXT |  |  |  |  |  |  |  |  |  | 1 |

Table 2: EXT = externalizing problem behavior. INT= Internalizing problem behavior. SDQ=Strengths and Difficulties Questionnaire. *Correlation is significant at the 0.05 level. **Correlation is significant at the 0.01 level.

sTABLE 3: Pearson’s correlations between the four polygenetic risk scores

|  | PRS Schizophrenia | PRS Depression | PRS Neuroticism | PRS Wellbeing |
| --- | --- | --- | --- | --- |
| PRS Schizophrenia | 1 | .352^**^ | .277^**^ | -.225^**^ |
| PRS Depression |  | 1 | .540^**^ | -.277^**^ |
| PRS Neuroticism |  |  | 1 | -.333^**^ |

Pearson’s correlations between polygenetic risk scores for Schizophrenia. Depression. Neuroticism and wellbeing*Correlation is significant at the 0.05 level. **Correlation is significant at the 0.01 level (2-tailed).

sTABLE 4: Results of the hierarchical regression analyses with the environmental risk factors and the PRSs for Schizophrenia. Depression. Neuroticism and Wellbeing predicting child internalizing and externalizing symptoms at ages 5/6 and 11/12.

|  | *PRS Schizophrenia* | | | | *PRS Depression* | | | | *PRS*  *Neuroticism* | | | | *PRS*  *Wellbeing* | | | |
| --- | --- | --- | --- | --- | --- | --- | --- | --- | --- | --- | --- | --- | --- | --- | --- | --- |
|  | β | R^2^ | ∆R^2^ | Pvalue ∆R^2^ | β | R^2^ | ∆R^2^ | Pvalue ∆R^2^ | β | R^2^ | ∆R^2^ | Pvalue ∆R^2^ | β | R^2^ | ∆R^2^ | Pvalue ∆R^2^ |
| Internalizing problems mother report age 5/6 |  |  |  |  |  |  |  |  |  |  |  |  |  |  |  |  |
| Model 1 (Child gender) |  | – | – | .625 |  | – | – | .625 |  | – | – | .625 | -.017 | – | – | .625 |
| Model 2 (+Environmental risk) |  | .040 | .040 | **<0.0001** |  | .040 | .040 | **<0.0001** |  | .040 | .040 | **<0.0001** |  | .040 | .040 | **<0.0001** |
| Model 3 (+Genetic risk) | .014 | .040 | – | .699 | .053 | .043 | .003 | .131 | .034 | .041 | .001 | .325 | -.027 | .041 | .001 | .442 |
| Externalizing problems mother report age 5/6 |  |  |  |  |  |  |  |  |  |  |  |  |  |  |  |  |
| Model 1 |  | .002 | .002 | .188 |  | .002 | .002 | .188 |  | .002 | .002 | .188 |  | .002 | .002 | .188 |
| Model 2 |  | .079 | .077 | **<0.0001** |  | .079 | .077 | **<0.0001** |  | .079 | .077 | **<0.0001** |  | .079 | .077 | **<0.0001** |
| Model 3 | .081 | .085 | .006 | .018 | .082 | .079 | – | 935 | -.020 | .079 | – | .553 | -.018 | .079 | – | .599 |
| Internalizing problems teacher report age 5/6 |  |  |  |  |  |  |  |  |  |  |  |  |  |  |  |  |
| Model 1 |  | .001 | .001 | .362 |  | .001 | .001 | .362 |  | .001 | .001 | .362 |  | .001 | .001 | .362 |
| Model 2 |  | .031 | .030 | **<0.0001** |  | .031 | .030 | **.003** |  | .031 | .030 | **.003** |  | .031 | .030 | **.003** |
| Model 3 | -.034 | .032 | .001 | .360 | -.034 | .032 | .001 | .357 | .004 | .031 | – | .919 | .007 | .031 | – | .851 |
| Externalizing problems teacher report age 5/6 |  |  |  |  |  |  |  |  |  |  |  |  |  |  |  |  |
| Model 1 |  | – | – | .744 |  | – | – | .744 |  | – | – | .744 |  | – | – | .744 |
| Model 2 |  | .025 | .025 | .011 |  | .025 | .025 | .011 |  | .025 | .025 | .011 |  | .025 | .025 | .011 |
| Model 3 | .017 | .026 | .000 | .658 | -.003 | .025 | – | .945 | -.006 | .025 | .000 | .877 | -.006 | .027 | .000 | .873 |
| Internalizing problems mother report age 11/12 |  |  |  |  |  |  |  |  |  |  |  |  |  |  |  |  |
| Model 1 |  | – | – | .821 |  | – | – | .821 |  | – | – | .821 |  | – | – | .821 |
| Model 2 |  | .117 | .117 | **<0.0001** |  | .117 | .117 | **<0.0001** |  | .117 | .117 | **<0.0001** |  | .117 | .117 | **<0.0001** |
| Model 3 | -.043 | .119 | .002 | .202 | .029 | .118 | .001 | .387 | .020 | .117 | – | .546 | .023 | .117 | .001 | .488 |
| Externalizing problems mother report age 11/12 |  |  |  |  |  |  |  |  |  |  |  |  |  |  |  |  |
| Model 1 |  | – | – | .822 |  | – | – | .822 |  | – | – | .822 |  | – | – | .822 |
| Model 2 |  | .113 | .113 | **<0.0001** |  | .113 | .113 | **<0.0001** |  | .113 | .113 | **<0.0001** |  | .113 | .113 | **<0.0001** |
| Model 3 | .005 | .113 | – | .875 | .011 | .113 | .000 | .741 | .040 | .115 | .002 | .229 | -.012 | .113 | .000 | .732 |
| Internalizing problems teacher report age 11/12 |  |  |  |  |  |  |  |  |  |  |  |  |  |  |  |  |
| Model 1 |  | .001 | .001 | .484 |  | .001 | .001 | .484 |  | .001 | .001 | .484 |  | .001 | .001 | .484 |
| Model 2 |  | .028 | .027 | .025 |  | .028 | .027 | .025 |  | .028 | .027 | .025 |  | .028 | .027 | .025 |
| Model 3 | -.068 | .032 | .005 | .101 | -.003 | .028 | – | .938 | -.022 | .028 | – | .604 | .032 | .029 | .001 | .435 |
| Externalizing problems teacher report age 11/12 |  |  |  |  |  |  |  |  |  |  |  |  |  |  |  |  |
| Model 1 |  | – | – | .647 |  | – | – | .647 |  | – | – | .647 |  | – | – | .647 |
| Model 2 |  | .054 | .054 | **<0.0001** |  | .054 | .054 | **<0.0001** |  | .054 | .054 | **<0.0001** |  | .054 | .054 | **<0.0001** |
| Model 3 | .047 | .057 | .002 | .245 | .015 | .055 | – | .709 | .027 | .041 | .001 | .513 | -.012 | .055 | – | .761 |
| Internalizing problems child’s report age 11/12 |  |  |  |  |  |  |  |  |  |  |  |  |  |  |  |  |
| Model 1 |  | .001 | .001 | .396 |  | .001 | .001 | .396 |  | .001 | .001 | .396 |  | .001 | .001 | .396 |
| Model 2 |  | .063 | .062 | **<0.0001** |  | .063 | .062 | **<0.0001** |  | .063 | .062 | **<0.0001** |  | .063 | .062 | **<0.0001** |
| Model 3 | .013 | .063 | – | .712 | .040 | .065 | .002 | .263 | .067 | .067 | .004 | .058 | -.043 | .065 | .002 | .229 |
| Externalizing problems Child’s report age 11/12 |  |  |  |  |  |  |  |  |  |  |  |  |  |  |  |  |
| Model 1 |  | – | – | .877 |  | – | – | .877 |  | – | – | .877 |  | – | – | .877 |
| Model 2 |  | .040 | .040 | **<0.0001** |  | .040 | .040 | **<0.0001** |  | .040 | .040 | **<0.0001** |  | .040 | .040 | **<0.0001** |
| Model 3 | .012 | .040 | – | .727 | .040 | .040 | .002 | .238 |  | .040 | .002 | .195 | -.035 | .039 | .001 | .323 |

Polygenetic risk scores that are reported here were calculated based on a Gaussian Prior of 1. Predictor in model 1:Gender. Predictors added in model 2: age mother at gestation. maternal smoking and use of alcohol during pregnancy. maternal education. history of psychopathology reported during pregnancy. maternal prenatal anxiety. maternal distress during measurement (age 5-6 or age 11-12). Predictors added in model 3: the PRS for either schizophrenia. major depression. neuroticism or wellbeing. β = standardised beta of the PRS; R^2^ = explained variance when all predictors are included in the model; ∆R^2^ =additional explained variance after including environmental risk factors (model 2) and the PRS (model 3). For none of outcomes of interest (model 3) we report significant findings after multiple correction (based on a p value of 0.004).
